# Perceptions and Experiences of Xylazine, Disparities in Xylazine Awareness, and Correlates of Xylazine-Attributed Wounds Among People Who Use Opioids

**DOI:** 10.3390/ijerph23010070

**Published:** 2026-01-02

**Authors:** Carl A. Latkin, Lauren Dayton, Haley Bonneau, Melissa A. Davey-Rothwell, Danielle German, Ananya Bhaktaram, Oluwaseun Falade-Nwulia

**Affiliations:** 1 Department of Health, Behavior and Society, Bloomberg School of Public Health, Johns Hopkins University, Baltimore, MD 21205, USA; 2 Division of Infectious Diseases, School of Medicine, Johns Hopkins University, Baltimore, MD 21205, USA

**Keywords:** xylazine, fentanyl, withdrawal, overdose, harm reduction, wound care

## Abstract

Background: Xylazine, an adulterant in the illicit opioid supply, heightens the risks of overdose, withdrawal severity, and severe wounds among people who use opioids (PWUO). Despite increasing prevalence, gaps remain regarding xylazine awareness in the drug supply and effective harm reduction interventions to address it. Methods: We conducted a cross-sectional survey of 703 PWUO in Baltimore, MD (2023–2025), to assess xylazine awareness, perceptions, and experiences. Multivariable logistic regression models examined correlates of xylazine awareness and self-reported xylazine-attributed wounds. Results: 84.8% of White participants, 48.6% Black participants, 64.3% of males, and 51.4% females had heard of xylazine. Nearly half (45%) of those who used xylazine reported that it caused more severe withdrawal symptoms. In the multivariable model of xylazine awareness, the largest odds ratios were year of survey administration (2024 vs. 2023: aOR = 4.30, 95% CI = 2.91–6.37; 2025 vs. 2023: aOR = 6.32, 95% CI = 3.31–12.07) and White race (aOR = 3.22, 95% CI = 1.85–5.57). Other significant demographic variables included education and gender. In the multivariable model of xylazine-attributed wounds, survey year 2025 vs. 2023 (aOR = 2.65, 95% CI = 1.06–6.61) and injection drug use in the prior year (aOR = 17.74, 95% CI = 5.58–56.39) were statistically significant. Conclusions: Awareness of xylazine in the drug supply remains incomplete among PWUO, with differences by race, age, and gender. The finding of a strong association of xylazine-attributed wounds and injection drug use should be the focus of future research. These findings underscore the need for enhanced surveillance systems, peer education, and community-based harm reduction strategies. Real-time monitoring and rapid response strategies are essential to protect against health risks of toxic adulterants, such as xylazine, medetomidine, and BTMP, in the drug supply.

## 1. Introduction 

Xylazine is a relatively recent adulterant in the opioid street drug supply, which was first detected in Puerto Rico in the early 2000s. Although it was present in toxicology of overdose deaths in Philadelphia, PA, USA, in 2006, it became more noticeable among people who use drugs in 2010 in Philadelphia and detected in 2016 in Baltimore, MD [1]. While xylazine was first identified in Puerto Rico and then the US mainland, it has now been detected in Canada, Australia, Europe, Mexico, and South America [2,3,4,5,6,7]. Xylazine, a veterinary sedative, is not approved for human use and is associated with fatal overdoses, blackouts, exacerbated withdrawal symptoms, and life-threatening soft tissue damage. Due to its respiratory and circulatory depressive effect, xylazine presents an increased risk for fatal overdose in combination with opioids. In overdoses resulting from opioids contaminated with xylazine, naloxone may counteract opioids but not the xylazine. There is no approved specific antidote in humans, and overdose management is primarily cardiovascular and respiratory support. Although clinical strategies to treat withdrawal symptoms from xylazine are emerging [8,9], there are no clinical trials for xylazine withdrawal alone or in combination with opioids. Another major public health issue is xylazine’s association with soft tissue wounds, which can become severe, especially if not treated early.

Several studies have examined the prevalence of xylazine in the drug supply. These rates have been found to vary significantly by location and date, ranging up to 98% in samples from Philadelphia, PA, USA [10]. The New York City Department of Health and Mental Hygiene’s drug checking program from samples collected at syringe service programs found xylazine in opioid samples increased from 10.7% in 2021 to 53.7% through 2024 [11]. The considerable variation in xylazine prevalence is likely due in part to different drug supply chains.

A statewide program in Maryland was established to test drug residue in paraphernalia returned to syringe service programs (SSPs) from 17 programs in 11 jurisdictions throughout the state [12]. Of the samples analyzed between October 2021 and October 2023, 33% contained xylazine. There were also significant differences across jurisdictions, with some counties finding that over 75% of samples contained xylazine, while others found concentrations of 10% or lower. In the first quarter of 2024, 38% of samples tested positive for xylazine. In the first quarter of 2025, this percentage decreased to 13%. The samples are voluntarily provided at the SSPs, and there is no associated sampling frame or way to know if the samples are unique. This high variability in xylazine positivity may make it highly difficult for people who use drugs to develop appropriate harm reduction strategies.

Studies have also examined xylazine awareness. In a study of hospital patients receiving treatment for opioid use disorder in New York State from September 2023 to August 2024, 92% reported awareness of xylazine [13]. Slightly over half (53%) of the participants believed that some of the drugs that they use contain xylazine, and 73.5% believed that exposure to xylazine increased their risk of overdose.

A study of people who use opioids (PWUO) in Lowell, Massachusetts, 2023 (*N* = 94), reported that half of the survey respondents were aware of xylazine [14]. A community-based survey from a convenience sample in Connecticut, between April 2021 and July 2022, found that most (68.5%) respondents (*N* = 667) were aware of xylazine in the drug supply [15]. Hispanic and White participants were significantly more likely to be aware of xylazine, but awareness of xylazine did not vary by type of drug use or route of consumption. A study of 94 PWUO in November-December 2022 in Baltimore, Maryland, found that 49% of respondents reported that they used drugs that were “cut with Xylazine sometimes known as tranq, tranq dope, or animal tranquilizer.” [16]. Few studies have examined factors associated with awareness of xylazine in the drug supply or how this has changed over time.

One of the most concerning effects of xylazine is wounds. There remains little information about factors linked to xylazine-caused wounds, which can appear in non-injection sites. Xylazine wounds can lead to secondary infections and necrosis requiring extensive surgical debridement and skin grafting, and in extreme cases, amputation [17,18,19,20,21,22,23]. Of the 171 respondents recruited from three SSPs in Massachusetts in August 2023, 87% (*N* = 148) reported a xylazine wound in the past 90 days [24]. There were no statistically significant demographic differences between those with and without xylazine wounds. However, among people who reported injecting drugs, skin popping (subcutaneous injection) was associated with xylazine wounds.

The goal of the current study was to examine (1) the prevalence and correlates of awareness of xylazine in the drug supply and changes in awareness over a 26-month period in Baltimore, Maryland, (2) the level of concern and experiences regarding xylazine among PWUO, and (3) the prevalence of wounds attributed to xylazine and factors associated with wounds.

## 2. Methods

Study Design and Participants: Participants were enrolled in the OASIS study, which examined geospatial factors associated with drug overdoses and trained individuals to distribute overdose and HIV/HCV prevention materials to settings where people use drugs. Community-based recruitment targeted areas with high drug use in Baltimore, Maryland. Eligibility criteria included (1) reporting illicit opioid use in the past 30 days, (2) age ≥18 years, and (3) residing in the Baltimore Metropolitan area. The Institutional Review Board at the Bloomberg School of Public Health approved the study protocols. Participants received $40 USD for completing the study visit. Between 4 April 2023 and 9 July 2025, 703 participants completed a baseline survey that contained xylazine-related questions. These questions were extensively piloted with participants.

### Measures

Outcome variables of xylazine awareness and wounds: Participants were first asked, “Have you ever heard about xylazine, which is also called ‘tranq’?” (Probe: A drug causing wounds). Affirmative responses triggered the administration of subsequent xylazine-related questions. Wounds were assessed with the question, “Have you had a wound that was caused by using xylazine/tranq?” (Yes/No/Don’t know).

Prevalence, perceived risk of exposure, and impact: Perceived xylazine prevalence in the community was assessed with the question, “What percentage of drugs on the streets of Baltimore do you think contain xylazine/tranq?” Response options included None (0%), Some (25%), About half (50%), Most (75%), All (100%), and Don’t know. Self-reported xylazine exposure was assessed by asking “Do you think you have used drugs containing xylazine/tranq?” (Yes/No/Don’t know). Participants reporting “Yes” were asked, “How does xylazine/tranq affect your withdrawal symptoms?” (Worse symptoms/No change/Fewer symptoms/Don’t know). To assess perceptions of the level of harm in the community, they were also asked, “How many people do you know have had wounds caused by xylazine/tranq?” Have you had a wound that was caused by using xylazine/tranq?

Xylazine-Related Concerns and Knowledge: Two items assessed concerns about xylazine: (1) “How worried are you about a drug buddy (friend) overdosing on xylazine/tranq?” and (2) “How worried are you about xylazine/tranq being in your drugs?” Response options were “Not at all worried, Just a little worried, Quite a bit worried, and Very worried.” Knowledge was assessed with the item “How effective is Narcan for an overdose caused by xylazine/tranq?” Response options included “Very effective, Somewhat effective, Not at all effective, and Don’t know.”

Substance Use Patterns and Medication for Opioid Use Disorder (MOUD): Participants reported on their history of using heroin, cocaine, crack, methamphetamine, buprenorphine, and fentanyl. Those reporting use of drugs within the prior three months answered frequency questions. For fentanyl: “In the past 3 months, how often did you use fentanyl? This includes using fentanyl alone or other drugs mixed with fentanyl.” Response categories for drug frequencies were “Never, Less than monthly, Monthly, Weekly, and Daily or Almost Daily.” Frequency categories were based on the European School Survey Project on Alcohol and Other Drugs (ESPAD) 2019 survey [25]. Drug use was dichotomized as high-frequency (daily or almost daily) versus lower-frequency use (less than daily or almost daily). Crack and powder cocaine use were combined into a single variable of cocaine use, and daily or almost daily use was categorized as high frequency.

Injection drug use, drug support, treatment, and use settings: Injection drug use was assessed with the question “When was the last time that you injected drugs?” with response options ranging from “Within the past day” to “Never.” Based on the distribution and the date when xylazine became prevalent in Maryland, those who reported injecting in the prior year were compared to all other participants. Other cut points that examined the last time injected yielded similar results. The number of drug use settings was measured by asking, “Thinking about the places that you use drugs, how many different places have you used in the past month (30 days)?” The responses were coded 1 through 10 and 11 or more. MOUD status was determined through questions about drug treatment history and current medication use. Participants reporting current use of buprenorphine/suboxone, methadone, naltrexone, or “other” medications for opioid use disorder were categorized as currently receiving MOUD. The variable that addressed drug-related social support was the question, “Do you have a running buddy? (A running buddy is someone that you hustle with, go in together with, and/or often use with).

Demographics: Self-reported demographics included age, race (African American/Black, White, Asian/Other/Refuse to answer), sex assigned at birth (male/female), and education (Grade 11 or less, Grade 12 or GED, Some college/Associate’s/Technical degree, and Bachelor’s degree or greater) was dichotomized to grade 11 or less and grade 12 or greater. Homelessness was defined as sleeping for at least one week in the past six months in non-traditional housing (squatting, abandoned building, car, shelter, park, or street).

Statistical Analysis: Primary outcomes were xylazine awareness (heard of) and xylazine-related wounds (ever). Temporal trends were examined by including the year of survey administration. Descriptive statistics and bivariate and multivariable logistic regression models were conducted. All demographic variables and others at the *p* < 0.15 level were included in the multivariable models. Odds ratios and 95% confidence intervals (CI) were calculated. Both calendar year and months since initial survey administration were modeled; given similar results, calendar year was retained for interpretability. Participants who reported knowing about xylazine were then asked if they believed that they had wounds from xylazine, and the second logistic regression model focused on these participants. Stata 17 (StataCorp LLC: College Station, TX, USA) was used for logistic regression analyses.

## 3. Results

The sample (*N* = 703) was primarily male (60.2%), Black (67.0%), with the majority reporting 12th grade education or higher (70.6%), and a mean age of 48.9 (Table 1). Almost half (47.8%) reported experiencing homelessness in the prior 6 months. Most participants (78.5%) reported daily or almost daily heroin and/or fentanyl use, about half (52.1%) daily or almost daily cocaine use, and more than a third (39.8%) had injected drugs in the prior year. Awareness of xylazine increased from 39.3% in 2023 to 83.7% in the first half of 2025.

Of 703 study participants, 416 (59.2%) reported awareness of xylazine, with 278 (67.0%) reporting that they had used drugs containing xylazine. There were 22.9% of study participants who reported that they did not believe xylazine was in their drugs, and 10.1% did not know (Table 2). Belief that xylazine was in participants’ drugs increased over time, from 58.4% in 2023 to 73.7% in 2025. There were 40.9% who reported that they thought xylazine was in some of the street drugs in Baltimore, while 23.6% estimated half, and 19.7% reported most. Few (7.0%) reported that it was in all their drugs, 1.4% of participants reported that they thought none of their drugs contained xylazine, and 7.5% responded that they “didn’t know.” Two-thirds (67.3%) of participants reported that they were “quite a bit” or “very” worried about a drug buddy or friend overdosing on xylazine, and the majority (74.5%) reported being quite a bit or very worried about xylazine in their drugs. Among the 278 participants who reported that they believed they had ever used xylazine, 44.6% reported worse withdrawal symptoms from xylazine; whereas 18.7% reported no change, 16.2% reported fewer symptoms, and 20.5% responded that they did not know. Roughly a third (31.6%) of participants reported not knowing anyone who had wounds caused by xylazine. However, the median number of friends in the study sample with xylazine wounds was 3, and the mean was 7.28 (SD = 14.05). Among those who believed that they had used xylazine, the prevalence of xylazine attributed wounds was 26.3% (Table 2).

### Bivariate Analyses

The logistic regression analyses examined factors associated with awareness of xylazine in the drug supply among all 703 study participants (Table 3). In the bivariate logistic regression analyses, there were several demographic factors associated with greater xylazine awareness, including higher levels of education (OR = 1.54, 95% CI = 1.11–2.14) and White and other races compared to Black (OR = 6.06, 95% CI = 3.92–9.35), (OR = 2.33, 95% CI = 1.18–4.62), respectively. Female sex was negatively associated with xylazine awareness (OR = 0.59, 95% CI = 0.43–0.80), as was older age (OR = 0.95, 95% CI = 0.94–0.96). Compared to participants interviewed in 2023, those interviewed in 2024 and 2025 had much greater odds, 5.21 (95% CI = 3.64–7.46) and 7.94 (95% CI = 4.39–14.38), respectively, of being aware of xylazine. Moreover, those who reported daily or almost daily heroin and/or fentanyl use were more likely to be aware of xylazine (OR = 1.69, 95% CI = 1.18–2.43), as were those who reported daily or almost daily cocaine use (OR = 1.47, 95% CI = 1.09–1.99) or reported injection drug use in the prior year (OR = 3.88, 95% CI = 2.76–5.44). A greater number of places of drug use in the prior month was also associated with xylazine awareness, with each additional drug use setting increasing the odds by 1.15 (95% CI = 1.10–1.20).

The second logistic regression model examined factors associated with self-reported wounds attributed to xylazine among the 278 participants who reported use of drugs they believed contained xylazine. In the bivariate models, White race, experiencing homelessness in the prior six months, daily or almost daily cocaine use, number of places used drugs in the preceding month, and survey year of 2025 compared to 2023 were significantly associated with xylazine attributed wounds. Of note, injection drug use in the prior year had an odds ratio of 17.25 (95% CI = 6.06–49.11) with reporting xylazine attributed wounds.

#### Multivariable Models

In the multivariable model for xylazine awareness, the largest odds ratios continued to be year of survey administration (2024 vs. 2023: aOR = 4.30, 95% CI = 2.91–6.37; 2025 vs. 2023: aOR = 6.32, 95% CI = 3.31–12.07), White race (aOR = 3.22, 95% CI = 1.85–5.57). A higher level of education (aOR = 1.54, 95% CI = 1.04–2.28) and female (vs. male) gender (aOR = 0.58, 95% CI = 0.40–0.85) also retained statistical significance in the multivariable model. The only other variable that remained statistically significant in the adjusted model was frequent heroin and/or fentanyl use (aOR = 1.60, 95% CI = 1.03–2.49). After adjusting for covariates, in the multivariable model for xylazine wounds, only the year 2025 vs. 2023 (aOR = 2.65, 95% CI = 1.06–6.61) for survey administration and injection drug use in the prior year (aOR = 17.74, 95% CI = 5.58–56.39) remained statistically significant.

## 4. Discussion

This study examined factors associated with xylazine awareness and wounds attributed to xylazine, as well as experiences and perceptions of xylazine. We found that the majority of participants were aware of xylazine. Awareness increased from 2023 to 2025 and was associated with male gender, White race, greater education, and more frequent opioid use. We also found a very large association between xylazine attributed wounds and injection drug use. One potential explanation for women reporting lower xylazine awareness compared to men is that women may be less likely to use drugs in public places, in part due to greater drug use stigma directed toward women compared to men. Safety concerns may also contribute to why women are less likely to report using substances in public places [26,27]. An ethnographic study of women who use drugs found that participants perceived the risk of violence to be the greatest when injecting in public and around acquaintances who were men [28]. Consequently, women may choose to remove themselves from public drug use settings due to safety or stigma related concerns. The indirect consequence of using in fewer public places is that women may be less likely to hear about changes in the drug supply—in this case, the proliferation of xylazine and its associated risks from other folks who are using in these settings. Women are also more likely to receive syringes and other harm reduction supplies from secondary sources, which may limit their direct access to new public health information [29]. Regardless of the reasons for the association, the findings suggest that outreach efforts on xylazine education should target women.

White individuals were more likely to be aware of xylazine than Black people. We do not know if this association is unique to Baltimore. In this sample, White PWUO appeared to be more likely to inject, be younger, and experience homelessness. This dynamic, linked to racial homophily, may lead White PWUO to observe more wounds and share information about xylazine. Additionally, as White PWUO were more likely to experience homelessness, they may be less able to care for and treat xylazine wounds, which could lead to more complex medical problems. There also may be greater connections, and hence drug information exchanged, among White PWUO in Baltimore to jurisdictions with higher prevalences of xylazine in the illicit drug supply. Frequent heroin/fentanyl use was also associated with xylazine awareness; this association may be due to more frequent interactions with other individuals who use drugs. The number of places where participants used drugs in the prior month was statistically significant in the bivariate but not the multivariable model, due in part to its strong association with injecting, high frequency heroin/fentanyl use, and experiencing homelessness.

Even though knowledge of xylazine has increased over time, it should not be assumed that all PWUO are knowledgeable about it or believe it is in the drug supply. Given the variability of knowledge, surveys should account for guessing and also assess the level of knowledge about drugs. However, the proportion of adulterants such as xylazine in the drug supply may change drastically in a short period, as can the number of adulterants in any batch. Moreover, other adulterants may exhibit similar features. This variability makes it difficult for people who use drugs to judge type types of adulterants or compounds, as well as the physiological impact and dangers of the drug. Xylazine drug test strips are critical for qualitative measures [30], but do not provide information on potency, nor do they provide guidance on methods to more safely use xylazine.

Several factors, including White race, experiencing homelessness, year of survey, number of places drugs were used, and history of injecting in the prior year, were significantly associated with having xylazine wounds in the bivariate analyses. However, in the multivariable models, only the variables of year of survey administration (2025 vs. 2023) and injecting in the prior 12 months were independently associated with wounds (aOR = 17.74). Those who did not inject in the prior year were ten times less likely to report a wound that was caused by xylazine (39.6% versus 3.6%). The magnitude of the association between injection drug use and wounds and the lack of plausibility of reverse causation suggest a causal association. However, several factors temper this conclusion. In addition to the cross-sectional study design, we do not know whether the wounds reported to be caused by xylazine were caused by xylazine. Wounds aggravated by bacterial or other pathogens could have been attributed to xylazine. However, it is also possible that some participants underreported wounds that were caused by xylazine because they did not attribute the wound to xylazine. We also did not assess the severity of the wounds or if they were the types of wounds typically attributed to xylazine. Moreover, as the rates of xylazine in the drug supply are highly variable, it is difficult to estimate the probability of exposure to xylazine. These findings should not be interpreted as indicating that non-injection ingestion of xylazine is safe, as we did not assess nasal damage caused by xylazine nor collect clinical data on wounds.

Other limitations include potential sampling biases, the lack of xylazine testing, and measures of preference for xylazine. However, Baltimore has had high variability in the proportion of drug samples that are positive for xylazine, and in qualitative interviews, study participants did not voice a preference for xylazine. This lack of preference may differ from cities such as Philadelphia, where the prevalence of xylazine has been high, and hence, people who use drugs may be more likely to develop xylazine-related drug dependence.

There were additional study limitations due to the limited survey items assessing xylazine. Researchers may want to consider additional methods to assess xylazine awareness and its associated harms. We do not know if study participants over- or under-reported xylazine awareness. To address underreporting, it can be valuable for harm reduction and public health programs to include ongoing qualitative interviews on people’s perceptions of types of street drugs, adulterants, and perceived impacts of these compounds. The lack of saturation of xylazine awareness in the community highlights the importance of peer education programs and trainings to diffuse information through the social networks of people who use drugs. Drug treatment programs, social services, emergency medical services, and healthcare providers can also provide information about xylazine and other harmful adulterants.

Reports of xylazine awareness may have been biased. A body of research suggests that people tend to overestimate their knowledge about a wide range of topics [31,32]. We do not know if this occurred in the present study regarding xylazine. One approach to addressing this bias in survey research is to include fictitious product names and ask respondents whether they are familiar with them. This approach could be applied to studying respondents’ knowledge of types of drugs circulating in the community.

It is also important to understand the sources of information, particularly information regarding novel drugs and adulterants, associated harms, and effective harm reduction techniques. We do not know how much information people may pass on from harm reduction organizations and other sources knowledgeable about the local drug supply to members of their social networks. We also need more ways to facilitate wound care and share best practices for effectively providing wound care to individuals who are reluctant or unable to obtain clinic-based medical care. As adulterants like xylazine continue to proliferate the drug supply, we need more inclusive measures beyond just overdose, such as wound severity, to assess the impact of the opioid epidemic on quality of life. Given the pain associated with wounds and impediments to receiving adequate pain management among people who use drugs, wounds may intensify opioid use, and healthcare providers need to provide options for pain management. Furthermore, even if individuals are able to access clinical care, for those who remain unstably housed, it may be difficult to keep wounds and dressings clean, leading to an increased likelihood of more complex problems such as gangrene and sepsis, leading to amputation.

Due to barriers to seeking medical care for wounds, future research should also explore alternative methods of documenting wounds, such as requesting individuals to photograph their wounds and send them to healthcare providers. Wounds may further exacerbate the already substantial stigma that people who use drugs face in hospital settings [33]. Compounded by delays in medical care due to transportation and competing priorities, we need methods such as telemedicine and street-based healthcare providers to provide real-time wound care.

As has been documented by other researchers, almost half (45%) of participants in the current study who experienced xylazine in their drugs reported more severe withdrawal symptoms [34]. Currently, there is limited information on effective harm reduction approaches to reduce withdrawal symptoms, and potential strategies should be investigated through qualitative and quantitative epidemiological and clinical research. Three-quarters of participants indicated that they were quite a bit or very worried about overdosing on xylazine. Hence, it would also be helpful to disseminate overdose response guidance that accounts for xylazine’s unique physiological effects.

Applied drug chemists and public health researchers and practitioners have developed methods to identify a wide range of drugs and compounds in samples obtained in the community [35,36]. These methods and systems of surveillance are critical. However, there is a need for chemists to develop quick and accurate test methods to reduce risks associated with illicit drugs.

Given the ability for illicit drug chemists to change the psychoactive compounds in illicit drugs, especially compared to previously, when street opioids were plant-based, we are already seeing that xylazine is being replaced in some jurisdictions by other harmful ingredients such as medetomidine and BTMPS [37]. Consequently, we need systems and methods of surveillance informed by people who use drugs and coupled with integrated lab and community-based approaches to harm reduction. Real-time surveillance systems also need to be linked to modeling based on distribution patterns to better inform local public health and harm reduction programs. Public health and harm reduction need to be able to quickly respond to variations in drug types and potencies. Drug treatment and healthcare programs also need to ensure that medications for treating drug dependence are of adequate doses with minimal side effects and that withdrawal symptoms for novel drugs can be managed. Moreover, overdose prevention and response programs need to be able to integrate effective methods of treating the involvement of novel drugs, such as xylazine, in opioid overdoses.

## Figures and Tables

**Table 1:** OASIS study participant characteristics, Baltimore, MD, 2023–2025 (*N* = 703).

Variables	Heard of Xylazine		
	Yes % (*N*)	No % (*N*)	Total % (*N*)
Sex at birth			
Male	64.3 (272)	35.7 (151)	60.2 (423)
Female	51.4 (144)	48.6 (136)	39.8 (280)
Race			
Black	48.0 (226)	52.0 (245)	67.0 (471)
White	84.8 (162)	15.2 (29)	27.2 (191)
Other	68.3 (28)	31.7 (13)	5.8 (41)
Education level			
Grade 11 or less	51.7 (107)	48.3 (100)	29.45 (207)
Grade 12/GED or more	62.3 (309)	37.7 (187)	70.55 (496)
Homeless, past 6 months	68.8 (231)	31.3 (105)	47.8 (336)
Current MOUD use	61.6 (215)	38.4 (134)	49.6 (349)
Daily, almost daily heroin/fentanyl (past 3 months)	62.0 (342)	38.0 (210)	78.5 (552)
Daily, almost daily cocaine (past 3 months)	63.7 (233)	36.3 (133)	52.1 (366)
Running buddy(ies)	62.5 (268)	37.5 (161)	61.0 (429)
Year of survey			
2023	39.3 (137)	60.7 (212)	49.6 (349)
2024	77.1 (202)	22.9 (60)	37.3 (262)
2025	83.7 (77)	16.3 (15)	13.1 (92)
Injection drug use (past 12 months)	77.5 (217)	22.5 (63)	39.8 (280)
Age in years (mean, SD)	46.52 (10.60)	52.27 (10.73)	48.87 (11.02)
Number of places where drugs were used (median, SD)	5 (3.67)	3 (3.24)	4 (3.60)

**Table 2:** Knowledge and perceptions of xylazine among OASIS study participants 2023–2025, Baltimore, MD.

Survey Question	Response Category	Percent
Have you ever heard about xylazine, which is also called “tranq?” (*N* = 703)	Yes	59.2
	No	40.8
Perceived percent of street drugs in Baltimore containing xylazine/tranq ^	None (0%)	1.4
	Some (25%)	40.9
	About half (50%)	23.6
	Most (75%)	19.7
	All (100%)	7.0
	Don’t know	7.5
Worried about a friend overdosing on xylazine/tranq ^	Not at all worried	10.8
	Just a little worried	21.9
	Quite a bit worried	26.9
	Very worried	40.4
Worried about xylazine/tranq in own drugs ^,*	Not at all worried	10.6
	Just a little worried	14.9
	Quite a bit worried	25.8
	Very worried	48.7
Can avoid xylazine/tranq if buying from a known dealer ^,*	Strongly agree	13.0
	Agree	24.8
	Disagree	37.8
	Strongly disagree	24.3
Perceived effectiveness of Narcan for xylazine/tranq overdose ^,*	Very effective	13.3
	Somewhat effective	21.9
	Not at all effective	37.1
	Don’t know	27.7
Think used drugs containing xylazine/tranq ^,*	Yes	67.0
	No	22.9
	Don’t know	10.1
Effect of xylazine/tranq on withdrawal symptoms ^$^	Worse symptoms	44.6
	No change	18.7
	Fewer symptoms	16.2
	Don’t know	20.5
Had a wound caused by xylazine/tranq use ^$^	Yes	26.3
	No	70.5
	Don’t know	3.2
Number of people they know that had wounds caused by xylazine ^,^@^	Mean	7.28 (SD = 14.05)
	Median	3

^—*N* = 416, ^$^—*N* = 278, *—missing data on one participant, ^@^—11 missing.

**Table 3:** Logistic regression models of knowledge of xylazine and wounds caused by xylazine.

Variables	Heard of Xylazine (*N* = 703)		Wound Caused by Xylazine (*N* = 278)	
	OR (95% CI)	aOR (95% CI)	OR (95% CI)	aOR (95% CI)
Education (Grade 12/GED or more vs. less)	**1.54** **(1.11–2.14)**	**1.54** **(1.04–2.28)**	1.07 (0.57–2.01)	1.15 (0.55–2.42)
Sex (female vs. male)	**0.59** **(0.43–0.80)**	**0.58** **(0.40–0.85)**	1.46 (0.83–2.55)	1.56 (0.78–3.10)
Age	**0.95** **(0.94–0.96)**	0.98 (0.96–1.00)	0.98 (0.95–1.01)	1.02 (0.98–1.06)
Race: White vs. Black	**6.06** **(3.92–9.35)**	**3.22** **(1.85–5.57)**	**2.73** **(1.49–4.98)**	1.01 (0.46–2.21)
Race: Other vs. Black	**2.33** **(1.18–4.62)**	2.07 (0.95–4.51)	0.23 (0.03–1.81)	0.17 (0.02–1.50)
Homelessness (past 6 months)	**2.16** **(1.59–2.95)**	1.00 (0.67–1.51)	**1.90** **(1.06–3.39)**	0.79 (0.37–1.67)
Current MOUD use	1.22 (0.90–1.65)	---	0.84 (0.49–1.44)	---
Daily, almost daily heroin/fentanyl (past 3 months)	**1.69** **(1.18–2.43)**	**1.60** **(1.03–2.49)**	1.51 (0.63–3.63)	---
Daily, almost daily cocaine use (past 3 months)	**1.47** **(1.09–1.99)**	0.90 (0.62–1.33)	**2.12** **(1.18–3.80)**	1.40 (0.70–2.78)
Running buddy(ies)	**1.42** **(1.04–1.93)**	1.11 (0.76–1.61)	0.94 (0.53–1.68)	**---**
Number of places where used drugs	**1.15** **(1.10–1.20)**	1.04 (0.98–1.10)	**1.12** **(1.04–1.21)**	1.07 (0.97–1.17)
Year of survey				
2024 (vs. 2023)	**5.21** **(3.64–7.46)**	**4.30** **(2.91–6.37)**	1.56 (0.78–3.13)	1.21 (0.55–2.65)
2025 (vs. 2023)	**7.94** **(4.39–14.38)**	**6.32** **(3.31–12.07)**	**3.18** **(1.45–7.01)**	**2.65** **(1.06–6.61)**
Injection drug use (past 12 months)	**3.88** **(2.76–5.44)**	1.51 (0.98–2.33)	**17.25** **(6.06–49.11)**	**17.74** **(5.58–56.39)**

OR—unadjusted odds ratio; aOR—adjusted odds ratio; Bold—statistically significant at *p* < 0.05.

## Data Availability

The data presented in this study are available on request from the corresponding author (The data are not publicly available due to ethical restrictions on sensitive information).
